# Crash characteristics and injury patterns among commercial motorcycle users attending Kitale level IV district hospital, Kenya

**DOI:** 10.11604/pamj.2014.19.296.4885

**Published:** 2014-11-17

**Authors:** Peter Kiteywo Sisimwo, Peter Kabanya Mwaniki, Christine Bii

**Affiliations:** 1 Jomo Kenyatta University of Agriculture and Technology, College of Health Sciences, Kenya; 2 Kenya Medical Research Institute, Centre For Microbiology Research, Nairobi, Kenya

**Keywords:** Crash characteristics, injury patterns, severity, commercial motorcycle, Kenya

## Abstract

**Introduction:**

Motorcycle users involved in crashes are likely to die or be severely injured due to high frequency of head, chest and leg injuries. We carried out a descriptive cross sectional study to determine crash characteristics and injury patterns among motorcycle users attending Kitale district hospital, Kenya.

**Methods:**

Motorcycle trauma patients were recruited between 1^st^ August 2013 and 31^st^October 2013. Data collection was done using a pre-tested, coded questionnaire. Frequencies mean (SD) and chi-square was employed in the analysis. Analysis was done using SPSS V.20. Results were considered significant at α = 0.05.

**Results:**

Motorcycle trauma patients formed 39.4% of all road traffic injuries. Males constituted 69.8%, females 30.2% and mean age was 30(±13) years. Riders accounted for majority of injury patients (45%), passengers (38.8%) and pedestrians (15.9%). Mechanism of motorcycle crash was involving motorcycle versus vehicle (45.6%). Riders suffered severe injuries compared to passengers (χ^2^=129.936, p < 0.001). Head injury patients were assessed as having Glasgow coma scale (GCS) of 70% 9-12, 26% GCS of 13-15 and 7% GCS of 3-8. Injuries sustained by victims included head and neck injury 40%, lower extremity injury 39.9% and chest injury 8.2%. Riders without helmets during the crash sustained head injuries (χ^2^=111.352, p < 0.001).

**Conclusion:**

Head injuries and lower extremity injuries accounted for the major proportion of injuries sustained by motorcycle users. Non helmet use was associated with increased risk of head injuries. Morbidity can be mitigated by encouraging use of protective gear like helmets.

## Introduction

Globally it is estimated that, 1.2 million people are killed in road crashes each year and as many as 50 million are injured [1]. With increasing modernization in many developing countries, road traffic deaths are increasing and traffic deaths are projected to become the third most important health problem by the year 2020 [2]. Motorcycle Injuries constitute a major but neglected emerging public health problem in developing countries and contribute significantly to the overall Road Traffic Injuries [1]. Motorcycle Injuries are among the leading causes of disability and deaths and the main victims are the motorcyclists, passengers and pedestrians in their young reproductive age group [3, 4]. The problem is increasing at a fast rate in developing countries due to rapid motorization and other factors [5]. The motorcycle, commonly called “bodaboda” in Uganda and Kenya [5, 6] and “Okada” in Nigeria [4, 7] has recently become increasingly popular in Kenya as a means of commercial transport. Their operation is characterized by non-helmet use by riders and passengers, passenger overload and lack of valid licensing among riders. Over speeding, reckless driving, lack of law enforcement and possible use of alcohol and drugs also characterise commercial motorcycle operations [8]. The scarcity of existing data on commercial motorcycle injuries in this environment despite morbidity and mortality resulting from motorcycle crashes necessitate a further look into the causative factors influencing the occurrence of such crashes. Studying the morbidity pattern of these motorcyclists will reveal the burden of the problem, as deaths and injuries due to road traffic crashes have not really been seen as a matter of public health importance. We carried out a descriptive cross sectional study of commercial motorcycle injuries presenting at Kitale district hospital to determine prevalence, severity and injury patterns among these patients.

## Methods

This was a descriptive cross-sectional study of patients with commercial motorcycle crash injuries of all age groups and gender presenting at the Accident and Emergency (A&E) department of Kitale level IV District Hospital. Kitale town is located at high Agricultural potential area of Trans-Nzoia County with an estimated population of 200,000. Kitale level IV district hospital provides Accident and Emergency services and its Government sponsored. Most patients seek health care services from the Government hospital due to lower charges as compared to the private hospitals. Trauma patients are first resuscitated and managed at the A&E Department according to the Advanced Trauma Life Support (ATLS) principles and then admitted to the admitting surgical firm. The study population comprised victims of commercial motorcycle crashes presenting at the Accident and Emergency department between 1^st^ August 2013 and 31^st^ October 2013. All victims of commercial motorcycle crash injuries presenting at the accident and emergency department were eligible for the study. At presentation, patients or their conveyers were interviewed by research assistants according to the details provided on a pro-forma. The data collected were patient's bio data, the injury sustained, mechanism of the crash injury, pre hospital transportation, body region injured, radiological findings, setting of the crash, condition of the road, collision type and use of helmet. The personal status during the crash was identified whether he/she was the rider, passenger and pedestrian. The fate of the subject whether discharged in good condition, admitted to the wards, referred or died was documented. Using the Fischer's exact test [9] the minimum sample size calculated was 384. The formula used for sample size determination was obtained as follows.

n = Z^2^_1-α/2_ PQ/d^2^

Where n = required sample size, α = level of significance (0.05), Q = 1-p, Z^2^_1-α/2_ = standard normal deviate within 95% confidence interval (1.96) P = assumed proportion of commercial motorcycle crash injuries among patients attending, Kitale level IV District Hospital (50%).

d = level of precision at 5% (standard value 0.05), n = (1.962×0.5×0.5) ÷ 0.052 n =384, Sample size = 384

But since the sampling frame was <10,000, the sample size was adjusted using the formula by fishers 1991, n= n/ (1 +n/N)

Where: n= initial sample size, N = sampling frame, n = new sample size, n= 384/ (1 +384/1800), = 317 motorcycle patients were required for the study.

Commercial Motorcyclists and passengers were eligible for inclusion in the study if they presented to the accident and emergency department within 24 hours of the Motorcycle crash injury. Motorcycle crash victims who came unconscious were also enrolled in this study after consent was obtained from their relative or from themselves after gaining conscious either in ICU or in the ward. Two trained research assistant were stationed in the accident and emergency department who interviewed patients and did data entry. Diagnosis was reached through clinical history, examination and radiological investigations. Topographic locations of injuries were then entered in the structured questionnaire. Information about diagnosis, registration number was retrieved from patient's file and admission register books. Data cleaning and validation was performed using SPSS version 20. Descriptive statistics such as mean, standard deviation, range and frequency proportions was performed. Pearson's chi square was used to test for the significance of association between dependent variable and independent variables. The level of statistical significance was set at P < 0.05. Binary logistic regression was used to adjust for confounding. Ethical approval was granted by the Kenya medical research institute ethics committee. The scientific steering committee of Kenya medical training institute also approved the study. Consent to carry out the study was also obtained from the administrators of the participating hospital.

## Results

**Figure 1:**
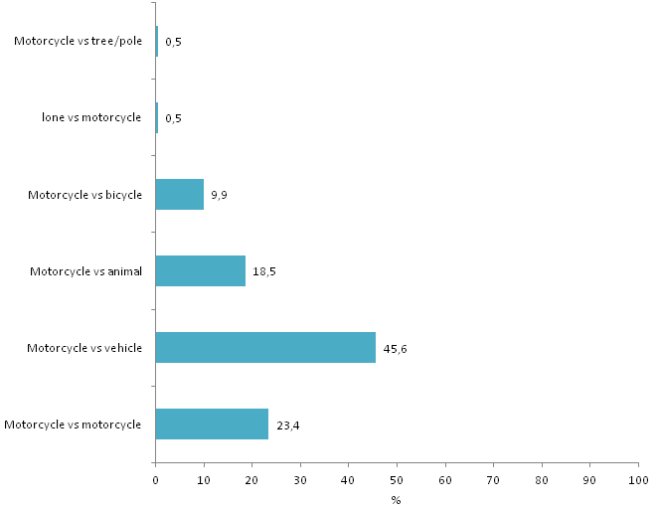
Mechanism of commercial motorcycle crash injury, Kitale, Kenya, 2013

During the study period 942 cases of road traffic injuries were seen at the Accident and Emergency department. Commercial Motorcycle traffic injuries accounted for 39.4% of all road traffic injuries. The mean age of the patients was 30.7 years with a standard deviation 0f 13. The youngest reported age was 3 years and the oldest was 80 years, with a range of 77 years. Majority of the patients were males 259 (69.8%) while females were 112 (30.2%), with a male to female ratio of 2.3:1. Most of the injured patients were riders 167 (45%), passengers injured accounted for 144 (38.8%), while pedestrians accounted for 59 (15.9%). Majority of the riders had attained primary level of education 106 (44%), while 92 (38.2%) of passengers and 43 (17.8%) of pedestrians had basic level of education (Table 1). Majority of the patients 348 (93.9%) reported highway as the place of injury crash. Only 1 (0.3%) occurred in rural roads. Most patients 314 (84.4%) were travelling at the time of injury crash and 47 (12.7%) were pedestrians. Motorcycle versus vehicle was the most reported mechanism of the motorcycle crash injuries 175 (45.6%). Motorcycle versus motorcycle was reported by 90 (23.4%) patients (Figure 1). Among the motorcycle crash injury patients, 167 (45.1%) were motorcycle riders while 144 (38.8%) were passengers.

**Table 1:** Socio demographic characteristics of respondents with commercial motorcycle crash injuries, Kitale, Kenya, 2013

Characteristics	Rider (%)	Passenger (%)	Pedestrian (%)	Total (%)
**Gender**				
Male	165 (64)	67 (26)	26 (10.1)	259 (69.8)
Female	2 (1.8)	77 (68.8)	33 (29.5)	112 (30.2)
**Religion**				
Christian	165 (45.5)	141 (38.8)	57 (15.7)	365 (98.4)
Muslim	1 (16.7)	3 (50)	2 (33.3)	6 (1.6)
**Marital status**				
Single	60 (38)	57 (36.1)	41 (25.9)	159 (42.9)
Married	107 (51.2)	84 (40.2)	18 (8.6)	209 (56.3)
Divorced	0(0)	2 (100)	0 (0)	2 (0.5)
Widowed	0(0)	1 (100)	0 (0)	1 (0.3)
**Education level**				
Primary	106 (44)	92 (38.2)	43 (17.8)	242 (65.2)
Secondary	61 (52.1)	40 (34.2)	16 (13.7)	117 (31.5)
College	0 (0)	12 (100)	0 (0)	12 (3.3)
**Occupation**				
Businessman/woman	1 (2.1)	31 (66)	15 (31.9)	47 (12.7)
Motorcyclist	161 (98.8)	1 (0.6)	1 (0.6)	163 (43.9)
Driver	0 (0)	3 (75)	1 (25)	4 (1.1)
Student	1 (1.6)	33 (51.6)	30 (46.9)	65 (17.5)
Farmer	1 (2.5)	35 (87.5)	4 (10)	40 (10.8)
Housewife	0 (0)	19 (79.2)	5 (20.8)	24 (6.5)
Other	3 (12)	21 (84)	1 (4)	25 (6.7)

Among the riders 49 (29.3%) suffered severe injuries as compared to passengers 9 (6.2%) and pedestrians 2 (3.4%). Passengers suffered moderate injuries 127 (88.2%) as compared to riders 106 (63.5%) and pedestrians 25 (42.4%). Pedestrians suffered minor injuries 32 (54.2%), riders 13 (7.7%) and passengers 8 (5.6%). This was statistically significant (χ^2^=129.936, p < 0.001) (Table 2). On arrival at the hospital, 240 (64.7%) patients with head injury were assessed as having Glasgow coma scale of between 9-12 moderate injury and 29 (7.8%) was between 3-8 severe injuries. Majority of the patients were treated as in-patients 317 (85.7%). Among those treated as outpatients, 38 (73.1%) were done minor surgery and 201 (63.8%) had major surgery. Hundred and forty seven (39.9%) had head and neck injuries and a similar proportion were injured in the lower extremity (Table 3). Majority of the riders who did not wear helmets at the time of crash suffered head injuries 89 (85.6%). Riders who wore helmets at the time of crash had no head injury 62 (98%) and this was statistically significant (χ^2^=111.352, P < 0.001) (Table 3).

**Table 2:** Relationship between category of road user and injury severity, Kitale, Kenya, 2013

Road user	Minor	Moderate	Severe	Totals
Rider	13 (7.7%)	106 (63.5%)	49 (29.3%)	168 (45.3%)
Passenger	8 (5.6%)	127 (88.2%)	9 (6.2%)	144 (38.8%)
Pedestrian	32 (54.2%)	25 (42.4%)	2 (3.4%)	59 (15.9%)
Totals	53 (14.2%)	258 (69.5%)	60 (16.1%)	371 (100%)

(c^2^=129.936, p < 0.001)

**Table 3:** Association between helmet use and Head injury among riders, Kitale, Kenya, 2013

Helmet use	Head injury	No head injury	Totals
Yes	1 (1.6%)	62 (98%)	63 (37.7%)
No	89 (85.6%)	15 (14%)	104 (62.3%)
Totals	90 (53.9%)	77 (46.1%)	167 (100%)

(c^2^=111.352, p < 0.001)

## Discussion

In this study the prevalence of Commercial Motorcycle crashes was in agreement with studies conducted in other developing countries. The reported prevalence of motorcycle injuries varies around the world, from 22.8% in China [10] to as high as 62% in Vietnam [11]. In Nigeria, the prevalence of motorcycle injuries varies from 12.8% -60% have been reported in different studies [12]. In Uganda the reported prevalence of boda boda injuries in Mulago hospital was 25% [6]. Differences in prevalence can be attributed to differences in risks such as age, sex, inappropriate speeds, use of helmets and appropriate pre hospital care. There is a male preponderance in this study which is in agreement with several other reports [6]. It is observed that nearly all commercial motorcyclists are males and riders constituted the single largest risk group. Other studies have identified riders as the majority of motorcycle crash victims presenting to hospitals [13]. High occurrences of motorcycles crashes among this group have been attributed to a wide range of activities engaged in by this class of people. They represent the active group that partake in high risk-taking activities such as recklessness riding, over-speeding and overloading their motorcycles, riding under the influence of alcohol and riding without wearing any protective gears. Males are more often exposed to traffic as drivers; they travel longer distances to work and are more often involved in use of automobile as leisure activities [14].

The commonest anatomical/site of injury were head and neck and lower extremities. The findings are similar to a study in Tehran, which documented that the commonest musculoskeletal injury was fracture of the tibia comprising almost 50% of cases [15]. Previous studies in Nigeria have shown that limb and head injuries are the commonest causes of morbidity and mortality in motorcycle injuries [7]. From a safety perspective a helmet is the most important part of a motorcycle. Its use has been shown to be 72% effective at reducing the incidence of head injuries [16]. In this study larger proportion of head injuries occurred among riders who did not wear helmets. Hence helmet use was a significant protective factor against head injury. Earlier studies have reported reductions in head injury associated mortality through the use of helmets [3]. The commonest cause of motorcycle crash was collision with a vehicle followed by collisions of motorcycle versus motorcycle. Vehicles have been reported to contribute majority of motorcycle crashes mainly due to their inability to detect or recognize them in traffic [4]. Similar findings have been reported elsewhere where up to 64% of motorcycle collisions were due to motorcycle and motor-vehicle collision [6]. In this study there was a significant relationship between the category of the road user and severity of the injury. Higher percentage of severe cases was among the motorcycle riders. However in a study of road traffic injuries in Western Maharashtra, no positive correlation existed between category of road user and severity of injury [17]. This could be attributed to differences in areas of study as well measurement scales of the study variable. Our study findings are subject to some limitations. We did not collect information from the first hospital the patient attended, it is possible that this would bias the interpretation of injuries sustained. Patients with minor injuries and never sought treatment were never captured. Pre hospital deaths were also not captured. In some instances it was impossible to corroborate independently the information provided by victims.

## Conclusion

Motorcycle crash is a major cause of road traffic injury among victims attended to at Kitale level IV district hospital. Male riders constituted the single largest risk group. Common cause of motorcycle crash was collision with a vehicle. Head injuries and lower extremity injuries accounted for major proportion of injuries sustained. Non helmet use was associated with increased risk of severe head injuries. Morbidity can be mitigated by use of protective gear like helmets and enforcement of traffic regulations.
